# Passive Anti-Amyloid Beta Immunotherapies in Alzheimer’s Disease: From Mechanisms to Therapeutic Impact

**DOI:** 10.3390/biomedicines12051096

**Published:** 2024-05-15

**Authors:** Thomas Gabriel Schreiner, Cristina Georgiana Croitoru, Diana Nicoleta Hodorog, Dan Iulian Cuciureanu

**Affiliations:** 1 Department of Medical Specialties III, Faculty of Medicine, “Grigore T. Popa” University of Medicine and Pharmacy, 700115 Iasi, Romania; 2 First Neurology Clinic, “N. Oblu” Clinical Emergency Hospital, 700309 Iasi, Romania; 3 Department of Electrical Measurements and Materials, Faculty of Electrical Engineering and Information Technology, Gheorghe Asachi Technical University of Iasi, 700050 Iasi, Romania; 4 Department of Immunology, “Grigore T. Popa” University of Medicine and Pharmacy, 700115 Iasi, Romania

**Keywords:** Alzheimer’s disease, amyloid beta, passive immunotherapy, monoclonal antibody, clinical trials

## Abstract

Alzheimer’s disease, the most common type of dementia worldwide, lacks effective disease-modifying therapies despite significant research efforts. Passive anti-amyloid immunotherapies represent a promising avenue for Alzheimer’s disease treatment by targeting the amyloid-beta peptide, a key pathological hallmark of the disease. This approach utilizes monoclonal antibodies designed to specifically bind amyloid beta, facilitating its clearance from the brain. This review offers an original and critical analysis of anti-amyloid immunotherapies by exploring several aspects. Firstly, the mechanisms of action of these therapies are reviewed, focusing on their ability to promote Aβ degradation and enhance its efflux from the central nervous system. Subsequently, the extensive history of clinical trials involving anti-amyloid antibodies is presented, from initial efforts using first-generation molecules leading to mixed results to recent clinically approved drugs. Along with undeniable progress, the authors also highlight the pitfalls of this approach to offer a balanced perspective on this topic. Finally, based on its potential and limitations, the future directions of this promising therapeutic strategy for Alzheimer’s disease are emphasized.

## 1. Introduction

Alzheimer’s disease (AD) is the most common type of dementia [1] and the most prevalent neurodegenerative disorder worldwide [2]. The latest epidemiological data show that over 6 million Americans and over 55 million people around the globe are diagnosed with AD, and these numbers are expected to dramatically increase in the following decades [3]. Additionally, AD poses a great burden at the populational level, with a significant negative impact on morbidity, disability, and mortality. From early symptoms such as memory deficits, language, and problem-solving impairment [4] to late-stage progressive cognitive deterioration and limitations in the activities of daily living (ADL) [5], AD significantly reduces the quality of life (QoL) of patients [6].

In spite of the fact that AD was first identified over a century ago by German psychiatrist and neuropathologist Alois Alzheimer [7], its exact cause remains unknown. Moreover, the underlying pathophysiological mechanisms responsible for disease progression are also merely partially understood [8]. In this context, several prominent hypotheses attempt to explain AD onset and evolution, with the cholinergic hypothesis [9], the neuroinflammation theory [10], the misfolded proteins hypothesis [11], and the oxidative stress theorem [12] being the most prominent. Among them, the amyloid cascade hypothesis [13] holds particular importance. On the one hand, the abnormal buildup of amyloid beta (Aβ) aggregates in the brain, from initially soluble oligomers to the final insoluble senile plaques, is a well-known hallmark of AD [14]. On the other hand, Aβ is closely related to different cellular and molecular mechanisms suspected to be involved in the pathogenesis of AD [15]. This explains the advancements in AD early diagnosis, Aβ being a significant biomarker widely used in clinical practice.

In addition to its implications in AD’s pathophysiology, the focus on Aβ is also relevant from a therapeutical point of view, as the amyloidogenic hypothesis has long been a prolific source for developing targeted therapies [16,17]. However, most anti-Aβ treatments were unsuccessful in clinical trials and never became available for daily clinical use [18]. Several possible explanations are considered: the unilateral focus of medication ignoring many relevant aspects of AD, the incomplete knowledge of the mechanisms involved in AD onset and evolution, or the insufficient monitoring of patients receiving experimental therapies [19]. Still, anti-Aβ drugs are a promising therapeutic avenue among the limited options of AD treatments, with proof being the fact that the Food and Drug Administration (FDA) recently approved Aβ-directed monoclonal antibodies, Aducanumab, although not without controversies [20], and Lecanemab [21].

Passive anti-amyloid immunotherapy remains a powerful therapeutic approach in AD, with an increasing number of experimental drugs being discovered and a considerable number of clinical trials being performed in recent years. This tendency is expected to continue in following years. On these grounds, this review aims to offer a critical and original analysis of the past, present, and in-development anti-amyloid immunotherapies. We have provided a detailed presentation of the mechanisms of action of these therapies, focusing on their ability to promote Aβ degradation and enhance its efflux from the central nervous system (CNS). Subsequently, we have presented the extensive history of clinical trials involving anti-amyloid antibodies, from initial efforts using first-generation molecules leading to mixed results to recent clinically approved drugs. Along with undeniable progress, we have also highlighted the pitfalls of this approach to offer a balanced perspective on this topic. Finally, based on its potential and limitations, the future directions of this promising therapeutic strategy for Alzheimer’s disease have been emphasized.

## 2. Amyloid-Cascade-Hypothesis-Based Therapies

Before outlining the myriad of anti-amyloid pharmacological therapies, a short revision of the amyloid cascade hypothesis is necessary. The current known role of Aβ as the central hallmark of AD is a consequence of several discoveries throughout the decades. First, the identification of amyloid plaques in an AD patient’s brain highlighted the relevance of this peptide in the pathogenesis of AD. Later, the discovery of inherited forms of AD linked to mutations in the gene for amyloid precursor protein (APP) supported the idea that abnormal processing and excessive accumulation of Aβ plays a crucial role in this disorder [22]. Further evidence related to other genes, such as presenilin and the one coding for apolipoprotein E, revealed how Aβ is produced and cleared from the CNS. Finally, despite not being completely understood, Aβ pathological CNS accumulation was closely correlated with other relevant processing supporting neurodegeneration, such as Tau protein aggregation [23] or chronic oxidative stress [24].

The amyloid cascade has been reviewed extensively in several studies [25,26], including the authors’ previous works [17,27]. In normal conditions, the amyloid precursor protein (APP) undergoes two-phase processing via the non-amyloidogenic pathway. Alpha (α)-secretase and gamma (γ)-secretase complexes are the two key enzymes modulating APP’s degradation. The soluble amyloid protein precursor alpha (sAPPα) fragment and the p3 peptide are the final products of the non-amyloidogenic pathway. While sAPPα is considered to be involved in several biologically relevant processes such as neuroprotection, memory formation, and synaptic plasticity [28], the role of the p3 peptide has not been completely determined [29].

Regarding the pathological processing of APP encountered in AD and other neurodegenerative disorders, Aβ is the final product of the amyloidogenic pathway. The non-physiological degradation of APP undergoes via a similar two-phase process, the first step being modulated by beta (β)-secretase, with beta-secretase 1 (BACE1) as the main β-secretase expressed in neurons [30]. Other enzymes, including BACE2 and cathepsin B, are also part of the β-secretase complex but with a much lower expression in neurons [31]. The second phase of the amyloidogenic pathway is modulated by the γ-secretase complex, similar to the non-amyloidogenic pathway. Finally, an aspect worth mentioning is related to the different isoforms of Aβ, with Aβ40 and Aβ42 being the major ones and the Aβ42/40 ratio being of high relevance as an AD biomarker [32]. The predomination of one isoform over the others is related to many factors, primarily the patient’s condition. In physiological circumstances, APP processing leads mainly to the production of Aβ40, a more soluble form of Aβ with suspected protective effects [33]. On the contrary, in pathological situations, such as AD, the production of Aβ42 is predominant, with this isoform having a high aggregation characteristic that leads to the formation of senile plaques [34].

**Figure 1:**
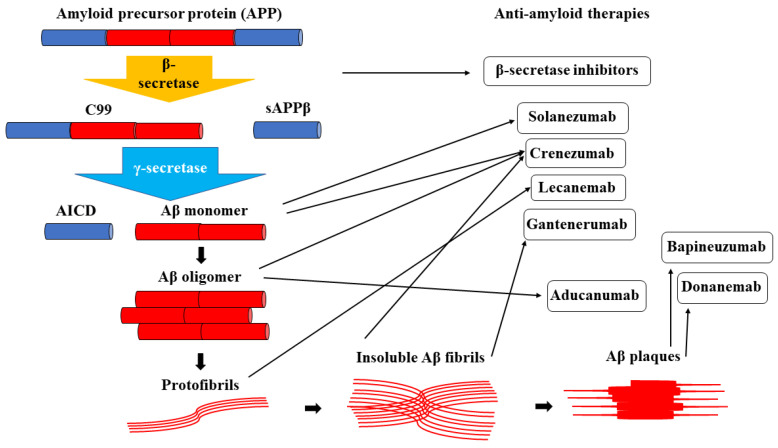
Anti-amyloid therapies targeting different phases of the amyloid cascade (designed based on a figure from Schreiner et al. [27]).

From a therapeutical point of view, the amyloid cascade hypothesis has been a prolific source of drug development, as schematically represented in Figure 1. Modulating the amyloidogenic pathway is not a new concept, as the first trials were conducted over 20 years ago. In the past, modulating Aβ production was considered the first logical therapeutic step to reduce Aβ cerebral load. In this regard, medication was designed to inhibit the main enzymes involved in pathological APP degradation. A broad group of pharmacotherapies is represented by BACE inhibitors, particularly BACE1 inhibitors. Despite BACE being the primary catalyst in APP metabolism, clinical trials based on BACE1 inhibitors have failed. Besides being inefficient in reducing Aβ cerebral load or improving clinical symptoms, drugs from this class also showed important side effects, such as accelerated brain atrophy and weight loss [35]. The administration of Atabecestat, another BACE1 inhibitor, was associated with a significant increase in serum liver enzymes, which is another relevant side effect [36]. Another noteworthy drug class is represented by Receptor for Advanced Glycation End Product (RAGE) inhibitors. RAGE is the primary influx transporter at the blood–brain barrier (BBB) level and modulates chronic neuroinflammation; thus, silencing RAGE was considered a promising therapeutic tactic. However, only one drug (Azeliragon) was tested in stage III clinical trial conditions, with research stopped early due to endpoint non-fulfillment [37].

The inefficiency of medication inhibiting Aβ production is mainly related to the fundamental mechanisms involved in the onset and evolution of sporadic, late-onset AD, based on impaired Aβ’s degradation and efflux from the CNS. In this regard, current therapeutic approaches aim to increase the forced elimination of Aβ from the CNS by indirectly lowering Aβ load in the periphery and the cerebrospinal fluid (CSF). The underlying mechanisms of the so-called “peripheral sink therapeutic strategy” and “CSF sink therapeutic strategy” have been reviewed in detail in other previous works [38,39]. Two distinct approaches, active and passive immunotherapies, aim to inactivate Aβ and favor its elimination. Active anti-Aβ immunotherapies were first explored in mouse models, where a reduction in Aβ cerebral load and decreased cognitive decline were observed [40]. However, the results are still unsatisfactory in humans. Worth mentioning is the human clinical trial involving AN1792, which was suspended early because of subacute meningoencephalitis in some patients [41]. To overcome this dangerous side effect attributed to uncontrolled activation of T and B lymphocytes, alternative adjuvants were tested in subsequent trials, along with several administration protocols and pathways (intranasal and subcutaneous). This strategy showed improvements in patients receiving anti-Aβ vaccines, who registered a reduced cognitive and functional decline, but no significant benefits related to brain volume loss [42]. However, future developments seem promising; for example, novel DNA-based vaccine technologies are more potent in improving dementia symptoms and are even being used as a prevention method in cognitively healthy persons or mild cognitively impaired individuals [43].

Passive anti-amyloid immunotherapies represent a complementary approach to active immunotherapies. This treatment modality has several advantages, such as greater dosage control and the possibility of withdrawing from treatment if any adverse events occur. The mechanisms behind passive immunotherapy and the most relevant drugs in clinical trials and daily use are further detailed.

## 3. Principles of Passive Anti-Amyloid Immunotherapies

Passive anti-amyloid immunotherapies are represented by monoclonal anti-Aβ antibodies produced in vitro and injected intravenously in AD patients. The beneficial effects of this therapeutic approach could be explained through several mechanisms observed in studies conducted in AD mouse models. Besides the straightforward effect of amyloid plaque removal from the mouse brain, the pro-inflammatory microglia were also modulated, according to earlier studies on the Tg2576 mice model [44]. The anti-Aβ antibodies exercise their function on the Aβ deposit after catalytic disruption of the amyloid plaque’s tertiary structure. Based on the “peripheral sink therapeutic strategy”, the anti-Aβ antibodies were designed to act upon the peripheral Aβ and not bind to the senile plaques located in the brain. This was demonstrated in studies conducted in mice when a significant increase in circulating plasma Aβ was observed after anti-Aβ antibody administration [45]. While the exact mechanisms related to Aβ-antibody complex clearance remain to be entirely determined, one major pathway in the CNS is related to microglia-dependent phagocytosis. The Fc-related mechanism is, however, doubled by local clearance that may occur in a non-Fc-mediated manner.

**Figure 2:**
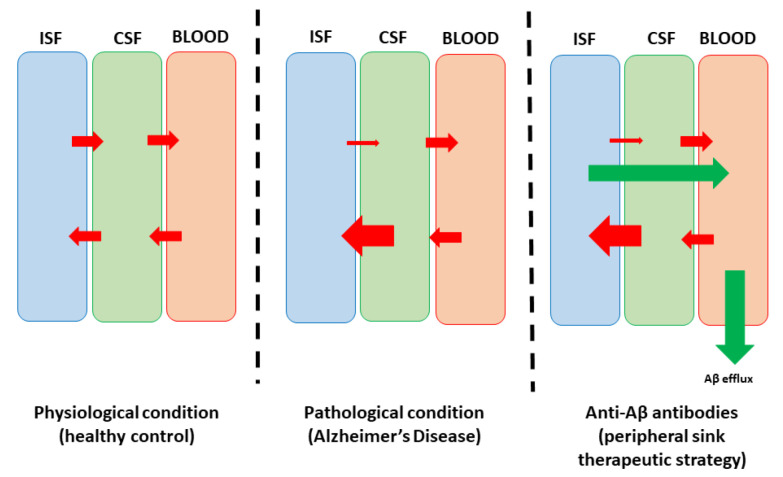
The “peripheral sink therapeutic strategy” explaining anti-Aβ antibody mechanisms of action. Abbreviations: ISF—interstitial fluid. CSF—cerebrospinal fluid. Red arrows—bidirectional flow of Aβ between the central and peripheral sinks. Green arrow—therapy-induced Aβ efflux (designed by modifying a figure from Schreiner et al. [39]).

Antibodies binding to plasma Aβ lead to the reduction of soluble Aβ plasma levels, which promotes the removal of soluble Aβ from the brain, with the BBB as the significant structure linking the brain parenchyma to the systemic circulation. Due to its structural and functional characteristics, the BBB remains a significant border in the bidirectional flow of larger molecules, including the anti-Aβ antibodies. The two major endogenous transport systems at the BBB level are receptor-mediated transcytosis and carrier-mediated transport. Still, receptor-mediated transcytosis, which involves the vesicular trafficking of ligand-receptor complexes, allows the delivery of larger molecules such as anti-Aβ antibodies. The transferrin receptor (TfR) has been extensively studied due to its high expression in brain endothelial cells. TfR-mediated transport utilizes the binding of antibodies to TfR, facilitating cargo transport across the BBB. Similarly, the insulin receptor (InsR) is another relevant target, with antibodies used as carriers to transport large proteins across the BBB. After being eliminated in the peripheral sink, the Aβ-antibody complex is eliminated via renal or hepatic mechanisms. Figure 2 highlights the “peripheral sink therapeutic strategy” explaining the mode of action of anti-Aβ antibodies.

To obtain effective anti-Aβ antibodies, extensive work has been conducted. Preclinical trials have tested several epitopes of the Aβ peptide (N-terminus, C-terminus, or the mid-region) to find the optimal IgG isotypes [46]. The undesired adverse effects were another relevant issue. One example is related to cerebral amyloid angiopathy-associated microhemorrhages and even larger acute hematomas that were observed in an N-terminal antibody administered to APP23 mice [47].

Despite significant advancements in the field of anti-Aβ antibodies, even the latest-generation drugs have their limitations. The first limiting aspect results from the criteria imposed by the FDA in the case of AD patients eligible for monoclonal antibody treatment. The need for compliance with clinical and paraclinical (CSF and imaging) biomarkers might limit the number of patients receiving these types of therapies, as was observed in the memory clinic setting (best-case scenario) where most of the patients did not meet the eligibility criteria for anti-amyloid treatment. While ensuring constant titers and allowing precise control with standardized perfusions, one major disadvantage of anti-Aβ monoclonal antibodies involves the economic aspect. The need for repeated (monthly or even more frequent) administration and the high single perfusion costs might be a critical restrictive aspect, particularly in developing countries [48]. From a clinical point of view, the limited research up to the present show that passive immunotherapy could alter the innate and adaptative immune systems in the direction of hyperactivation. Toll-like receptor 4 (TLR4) seems to be a link between Aβ and microglia activation [49], while there is still debate on the crosstalk between T cells and members of the amyloid cascade [50]. Based on magnetic resonance imagery (MRI) studies of the brain, anti-Aβ antibodies can cause amyloid-related imaging abnormalities (ARIA) in patients receiving this kind of therapy. The effect of ARIA-E (cerebral edema) and ARIA-H (cerebral microhemorrhages) depends on the severity and location of the imagery abnormalities [51]. According to their characteristics, ARIA can be asymptomatic; might be associated with nonspecific symptoms such as headache, nausea, vomiting, or confusion; or may lead to focal neurological signs, vertigo, mental state changes, and gait disturbances [52]. Drug-related ARIA modifications impose periodic MRI follow-ups, meaning extra financial costs for the individual and the healthcare system.

**Figure 3:**
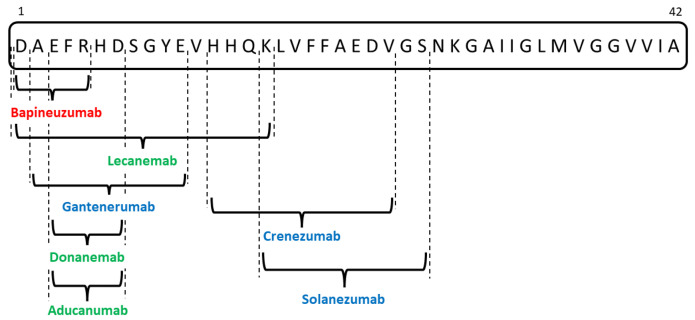
Overview of the anti-Aβ monoclonal antibodies targeting different epitopes of the Aβ42 peptide (red—first-generation medication; blue—second-generation drugs; green—third-generation drugs).

The group of passive anti-amyloid immunotherapies comprises a heterogeneous assembly of different-generation anti-Aβ monoclonal antibodies, two of which were recently approved for daily clinical use in AD patients. These drugs form a heterogeneous group, each targeting a different domain of the Aβ molecule, as depicted in Figure 3. Further, the most significant clinical trials on this topic are presented, focusing on the most discussed (and controversial) molecules over the last few years.

## 4. Clinical Trials and Relevant Drugs

Due to the considerable prior preclinical work, the anti-Aβ passive immunotherapy repertoire has now been extended to target several Aβ species, including monomers, oligomers, protofibrils, and insoluble plaques. Bapineuzumab was the first-generation monoclonal antibody directed towards the aggregated Aβ’s N-terminus [53]. Subsequently, second-generation medications targeting monomeric and fibrillary Aβ species, such as Crenezumab, Gantenerumab, and Solanezumab, were developed [54]. The third-generation monoclonal antibodies include monoclonal antibodies with high affinity against Aβ protofibrils (Lecanemab) [55] or senile plaques (Aducanumab [56], Donanemab [57]).

Bapineuzumab is an anti-3D6 humanized antibody that was directed against residues 1–5 at the Aβ protein’s N-terminus [53]. The drug’s unique specificity renders it unable to recognize unprocessed APP, while the 3D6 epitope can be found in several Aβ species, ranging from soluble oligomers to compacted Aβ plaques. The mechanisms of neutralizing and eliminating cerebral Aβ rely on the stimulation of Fc-receptor-mediated microglial phagocytosis [58]. Still, according to a phase 3 study comprising 1331 mild to moderate AD patients, Bapineuzumab showed no significant improvement compared to a placebo in terms of primary cognitive outcome [59]. This unfavorable outcome might be due to the drug’s poor efficacy in removing Aβ plaques or due to AD progression despite plaque clearance. The trial has its limitations, as no brain PET amyloid imaging was employed to enroll patients, as this method was not commonly accessible at that time. This is important considering recent research that points towards the importance of PET imaging in AD diagnosis, with up to 50% of clinically diagnosed dementias being Aβ brain negative in PET [60].

Second-generation anti-Aβ monoclonal antibodies are a heterogeneous group, with different affinities toward different Aβ species. Solanezumab exclusively targets soluble monomeric Aβ as it recognizes a linear epitope of the mid-domain (residues 16–26) Aβ molecule, which is undetectable in oligomers and fibrils [61]. Solanezumab’s mechanism is based on reducing synaptic toxicity and promoting Aβ movement from the central to the peripheral sink. Despite being well tolerated in phase 3 clinical trials in mild and moderate AD patients [62], with no incidence of ARIA, the drug showed no benefit on cognitive function when compared to a placebo. Additionally, Solanezumab was examined in people with pre-symptomatic AD who had a positive brain amyloid PET scan. In this trial, the drug showed no effect on cognitive decline, with Aβ building up over time, similar to the control group [63].

Gantenerumab targets insoluble fibrillar Aβ, exhibiting affinity for the N-terminal to the mid-domain region of Aβ, encompassing residues 3–11 and 18–27 [64]. Fibrillar Aβ inhibition is possible via phagocytosis and glial activation. Unfortunately, the clinical trials showed no beneficial effect on cognitive decline; moreover, the dose-dependent increase in ARIA incidence was another reason for the premature termination of trials [65]. Studies on mild cognitive impairment (MCI) and mild AD patients also missed the primary clinical endpoints. At the same time, Tau PET imaging showed no impact of the drug on Tau protein cerebral accumulation [66].

Crenezumab, a completely humanized monoclonal antibody, has a tenfold greater affinity for oligomers than monomers despite binding to all types of Aβ species. Its mechanism is based on regulating phagocytosis while lowering the production of inflammatory cytokines from microglia and complement activation [67]. According to a phase 2 trial, Crenezumab was most beneficial for early disease, reducing cognitive decline in MCI, mild AD, and mild-to-moderate AD patients, with a high suitability for intravenous administration [68]. However, two phase 3 studies on moderate AD subjects concluded that Crenezumab did not reverse cognitive impairment [69], while a trial in cognitively unimpaired members with presenilin 1 mutation did not reach its primary and secondary endpoints, leading to the suspension of drug development [70].

Third-generation anti-Aβ monoclonal antibodies are the newest and most successful passive immunotherapy medications, two of which are already FDA-approved for clinical use. Aducanumab, targeting residues 3–7 in the Aβ N-terminus, is highly selective for oligomeric or fibrillar aggregates due to its significant avidity for epitope-rich aggregates and poor monovalent affinity [71]. Preclinical studies showed a dose-dependent decrease in all types of Aβ deposits in the cortex and hippocampus when Aducanumab was administered in AD animal models, opening the pathway for several phase I and II clinical trials [72]. Of interest for the drug’s clinical approval remain the two multicenter, double-blind, randomized, placebo-controlled phase 3 trials, EMERGE (*n* = 1638) and ENGAGE (*n* = 1647), conducted on MCI and mild dementia patients. In the EMERGE trial, the primary clinical outcome was achieved, with 10 mg/kg Aducanumab leading to a 22% reduction in clinical progression and less cognitive decline [73]. On the other hand, no differences were seen in primary or secondary outcomes between the treatment and the placebo groups in the ENGAGE trial, indicating that clinical outcomes were not reached [74]. Still, in both trials, a significant reduction in brain Aβ from baseline was demonstrated based on the amyloid PET results [74]. Considering the side effects, both ARIA-E and ARIA-H were common findings, the majority being asymptomatic. Regarding the low incidence of symptomatic ARIA, patients complained of mild symptoms, with headache as the most common [75]. With one study meeting the endpoints and the other failing to demonstrate efficacy, this discrepancy in clinical outcomes sparked an explosive debate related to the fast-forward approval of Aducanumab by the FDA in June 2021 as the first disease-modifying therapy for AD [76]. Its use is often advised for individuals with clinical traits resembling those of patients who participated in the EMERGE and ENGAGE trials, as well as for those with positive AD biomarkers [77]. To lower the risk of ARIA, oral anticoagulant status, baseline cerebral amyloid angiopathy load measured using MRI, and careful evaluation of the apolipoprotein E (APOE) genotype should be conducted before selecting eligible patients and initiating Aducanumab. Currently, two ongoing trials aim to investigate Aducanumab’s real-world safety. On the one hand, the phase 3β EMBARK trial assessed the safety and tolerability of intravenous monthly use of 10 mg/kg of Aducanumab in patients who participated in previous Aducanumab studies. On the other hand, the ongoing phase 4 ADUHELM ICARE AD-US trial, proposed to enroll roughly 6000 persons, was designed to produce data on long-term and validate clinical outcomes [78]. As for daily clinical use, most recent reports suggest that the discontinuation of Aducanumab will occur in 2024, focusing on other monoclonal antibodies, such as Lecanemab.

Lecanemab, a humanized monoclonal antibody with remarkable selectivity against Aβ monomers and insoluble fibrils, is the second FDA-approved, AD-disease-modifying therapy for clinical use [79]. A phase 1 trial assessed the drug’s safety and tolerability in mild to moderate AD patients who received a four-month course of Lecanemab, with little occurrence of ARIA or asymptomatic ARIA-H being detected [80]. Subsequently, Lecanemab was evaluated in an 18-month multicenter phase 2b placebo-controlled trial conducted on MCI and mild AD dementia to determine the optimal dose and maximize the drug’s efficiency [81]. Clarity AD is the main clinical trial that offered relevant data for later drug approval in January 2023. In this multicenter, double-blind, randomized, placebo-controlled, parallel-group phase 3 study, Lecanemab was administered as 10 mg/kg biweekly in MCI or mild Alzheimer’s dementia patients, resulting in a more significant reduction in Aβ burden compared to a placebo [82]. The inevitable ARIA incidence (both symptomatic and asymptomatic ARIA-E and ARIA-H) was higher in the group receiving monoclonal antibody treatment; however, it was considered acceptable for the FDA accelerated approval for daily clinical use [83]. Currently, extensions of the previous studies or new clinical trials such as AHEAD 3-4/5 are evaluating the impact of long-term administration of Lecanemab on cognitive function and Aβ brain load, with final results to be available in the next few years [84].

Donanemab, another humanized monoclonal antibody discussed in this review, targets the Aβ’s N-terminal pyroglutamate p3–7 epitope found mainly in aggregated Aβ [85]. TRAILBLAZER-ALZ, a phase II clinical trial, demonstrated the beneficial but limited impact of Donanemab on the reduction of cognitive and functional decline [86]. The clinical effects were doubled in the PET imaging results, which showed a substantial decrease in amyloid plaque levels by week 76 of treatment, with more than 50% of the subjects achieving amyloid-free status after 52 weeks [86]. Additionally, a post hoc analysis for Donanemab-treated participants suggests an indirect effect of slowing Tau protein accumulation, highlighting the intricate mechanisms of the disease [87]. Still, the incidence of ARIA is a common problem, being noted in 40% of Donanemab-treated patients, with 26% displaying symptomatic ARIA. Donanemab showed further encouraging results in phase III trials conducted on MCI and mild AD dementia patients. The primary endpoint was met, with a 35% reduction in cognitive and functional decline being observed; the secondary endpoints revealed a similar trend, with slowed deterioration and a lower rate of AD progression [88]. Similar to other third-generation monoclonal antibodies, selecting adequate patients, such as considering their APOE ε4 status, for Donanemab administration is essential, particularly when considering the risk of developing symptomatic ARIA [89]. Table 1 summarizes the most relevant aspects of monoclonal antibodies previously discussed.

Finally, there are three other monoclonal antibodies currently in the development phase. ACU193 is an IgG2 humanized monoclonal antibody capable of selectively binding to soluble Aβ oligomers. Currently, ACU193 is evaluated in a multicenter phase 1 clinical trial comprising MCI and mild AD individuals [90]. This was a natural step, considering the preclinical studies highlighting the significant benefits of ACU193 when administered in transgenic mice. The drug positively impacted hyperactivity and cognitive performance in AD mice during pre- and post-amyloid plaque deposition phases. Another promising candidate is trontinemab, a bispecific monoclonal antibody specifically engineered to bind monovalently to the human transferrin receptor 1 and bivalently to Aβ plaques [91]. This drug is an updated variant of Gantenerumab, with improved blood–brain barrier penetration and similar efficacy at lower doses compared to Gantenerumab [91]. Similar to ACU193, trontinemab is included in a phase 1 clinical trial, including early phase AD patients, with results expected in the next few years. Lastly, remternetug, a humanized IgG1 antibody, was specially designed to target the amyloid precursor protein (APP) Aβ42’s N3pGlu peptide. The drug binds exclusively to the brain Aβ by selectively targeting the alteration of pyroglutamate. Currently being tested on participants with MCI or mild AD in a phase 1 clinical trial, initial results show a successful shift from PET-positive to PET-negative cerebral Aβ load [92]. With a phase 3 study in early symptomatic AD patients also ongoing, the near-future advancements are promising.

The discussion on anti-Aβ monoclonal antibodies would not be complete without a summary of these drugs’ side effects. During the different phases of clinical trials, Bapineuzumab showed mild to moderate adverse events, such as headache, back pain, anxiety, and fatigue, but also more severe ones, with vasogenic edema reported in both *APOE* ε4 and non-*APOE* ε4 patients [58]. Regarding second-generation medication, Solanezumab was associated with four types of adverse events, which occurred more frequently compared to a placebo, namely vitamin D deficiency, spinal osteoarthritis, nasal congestion, and dysuria [62]. Patients treated with Gantenerumab showed local adverse effects, mainly injection site erythema (in under 15% of the studied cohort), and CNS-related side effects, such as ARIA-E and ARIA-H [64]. When considering Crenezumab, 94% of patients receiving this drug reported at least one adverse event, the majority being mild or moderate [93]. There were no ARIA-E occurrences noted, but, in 10% of participants, new ARIA-H was recorded [93]. Symptomatic and mostly asymptomatic ARIA-E and ARIA-H could be commonly found in third-generation anti-Aβ monoclonal antibodies, as previously described, explaining the need for regular MRI follow-up during treatment. Other nonspecific side effects such as headache, nausea, dizziness, confusion, cough, or gastrointestinal were also mentioned. Still, larger cohorts of randomized clinical trials and real-world data are necessary to adequately evaluate the impact of adverse effects of Lecanemab and Donanemab.

## 5. Future Perspectives and Conclusions

The promising results of clinical trials related to the development of anti-Aβ human monoclonal antibodies for AD brings renewed hope for dementia patients and in a domain where no effective therapies exist, despite intensive research. It took three generations of passive anti-amyloid therapies to achieve the first accelerated, FDA-approved drug, Aducanumab, and the real-world data are still significantly less encouraging compared to preclinical expectations. With many ongoing studies, the first almost immediate aspect is related to the termination of phase-three trials. Another relevant aspect concerns the conclusions drawn from already finished clinical research. While many showed a reduction in PET-measured cerebral Aβ load, only a few doubled these impressive results and showed clinical improvement. This debatable aspect must be investigated in future studies, including research focused on a better understanding of the crosstalk between Aβ pathology and other relevant aspects of AD, such as Tau protein accumulation, neuroinflammation, and oxidative stress, as has already been pointed out in several works [94,95]. Even Aducanumab, which showed contradictory clinical results in the EMERGE and ENGAGE trials, received temporary approval, highlighting the importance of rethinking the criteria that AD monoclonal antibodies should pass before entering daily clinical use. Ethical dilemmas regarding the amyloid status of subjects included in research and future patients eligible for antibody-based treatment should also be addressed, particularly those with no absolute paraclinical biomarkers for AD monitoring.

Minimizing adverse effects is another critical treatment-dependent aspect worth considering, with symptomatic ARIA as the main concern for both patients and clinicians. This is highlighted in the context of the new hypothesis regarding ARIA as an inflammatory phenomenon triggering the complement cascade, resulting in damage to the cerebral vessel walls [51]. Moreover, the optimization of anti-Aβ monoclonal antibody delivery should also be considered. As discussed previously, the BBB significantly lowers drug penetration into the CNS, identifying this structural and functional barrier as being one of the current concerns in pharmaceutical research. Alternative drug delivery pathways, such as intranasal or intrathecal administration, could be considered in the near future. The financial burden on patients resulting from the periodic administration of expensive anti-amyloid drugs should be considered, particularly in the healthcare systems of developing countries.

With increasing prevalence and incidence worldwide, AD remains a challenge in terms of effective treatment. In this context, anti-amyloid monoclonal antibodies are a powerful therapeutic modality, with the growing number of clinical trials and different drugs in recent years indicating a promising breakthrough in the near future. Three generations of anti-Aβ antibodies have been designed, with only two drugs (Aducanumab and Lecanemab) having received clinical use approval. Still, encouraging preliminary results suggest Donanemab to be the next drug of this class available for early symptomatic AD patients. Finally, other molecules in different testing phases should be closely monitored to ensure future anti-AD medication is more efficient and safer for large-scale clinical use.

## Figures and Tables

**Table 1:** Most relevant characteristics and clinical trials on anti-Aβ monoclonal antibodies.

Anti-Aβ Monoclonal Antibody	Generation	Targeted Aβ Species	Relevant Clinical Trials	Current Status
Bapineuzumab	First generation	Aβ plaques	NCT00575055NCT00574132	Endpoints not met
Solanezumab	Second generation	Aβ monomers	EXPEDITION DIAN-TU	Primary points not met
Gantenerumab	Second generation	Insoluble Aβ fibrils	Scarlet RoAD Marguerite RoAD GRADUATE	Ongoing trials
Crenezumab	Second generation	Aβ monomers Aβ oligomers Insoluble Aβ fibrils	ABBY BLAZE CREAD	Primary and secondary points not met
Aducanumab	Third generation	Aβ oligomers	EMERGE ENGAGE	FDA approved
Lecanemab	Third generation	Protofibrils	Clarity AD AHEAD 3-4/5	FDA approved
Donanemab	Third generation	Aβ plaques	TRAILBLAZER-ALZ	Waiting for FDA approval

## Data Availability

Data are contained within the article.
